# Expression of MicroRNA-15b and the Glycosyltransferase GCNT3 Correlates with Antitumor Efficacy of Rosemary Diterpenes in Colon and Pancreatic Cancer

**DOI:** 10.1371/journal.pone.0098556

**Published:** 2014-06-03

**Authors:** Margarita González-Vallinas, Susana Molina, Gonzalo Vicente, Virginia Zarza, Roberto Martín-Hernández, Mónica R. García-Risco, Tiziana Fornari, Guillermo Reglero, Ana Ramírez de Molina

**Affiliations:** 1 Unit of Molecular Oncology and Nutritional Genomics of Cancer, Madrid Institute for Advanced Studies on Food (IMDEA-Food), Campus of International Excellence UAM+CSIC, Madrid, Spain; 2 Department of Production and Characterization of Novel Foods, Institute of Food Science Research (CIAL), Campus of International Excellence UAM+CSIC, Madrid, Spain; Universidad de Malaga, Spain

## Abstract

Colorectal and pancreatic cancers remain important contributors to cancer mortality burden and, therefore, new therapeutic approaches are urgently needed. Rosemary (*Rosmarinus officinalis* L.) extracts and its components have been reported as natural potent antiproliferative agents against cancer cells. However, to potentially apply rosemary as a complementary approach for cancer therapy, additional information regarding the most effective composition, its antitumor effect *in vivo* and its main molecular mediators is still needed. In this work, five carnosic acid-rich supercritical rosemary extracts with different chemical compositions have been assayed for their antitumor activity both *in vivo* (in nude mice) and *in vitro* against colon and pancreatic cancer cells. We found that the antitumor effect of carnosic acid together with carnosol was higher than the sum of their effects separately, which supports the use of the rosemary extract as a whole. In addition, gene and microRNA expression analyses have been performed to ascertain its antitumor mechanism, revealing that up-regulation of the metabolic-related gene GCNT3 and down-regulation of its potential epigenetic modulator miR-15b correlate with the antitumor effect of rosemary. Moreover, plasmatic miR-15b down-regulation was detected after *in vivo* treatment with rosemary. Our results support the use of carnosic acid-rich rosemary extract as a complementary approach in colon and pancreatic cancer and indicate that GCNT3 expression may be involved in its antitumor mechanism and that miR-15b might be used as a non-invasive biomarker to monitor rosemary anticancer effect.

## Introduction

Colorectal cancer is the third most diagnosed cancer type in males and the second in females worldwide, and its incidence is increasing even in traditionally low-risk countries such as Spain [1]. Moreover, mortality rates caused by colorectal cancer remain high, being the fourth and third cause of cancer-related mortality in males and females, respectively [1]. On the other hand, pancreatic cancer affects 250 000 individuals worldwide annually [2]. Although its incidence rates are not very high, it is one of the most lethal tumors, representing the five and fourth cause of cancer-related mortality in males and females, respectively, in developed countries [1]. Therefore, new complementary therapeutic approaches, ideally cost-effective and non-toxic, are needed to improve efficacy and quality of life of patients with these cancer types.

Rosemary (*Rosmarinus officinalis* L.) and many of its components were reported to possess chemopreventive properties in skin [3] and breast [4] cancers *in vivo*, mostly by inhibiting 7,12-dimethylbenz(a)anthracene (DMBA)-DNA adduct formation. Moreover, they exert antioxidant activity both *in vitro* [5], [6] and *in vivo* [7], thus inhibiting genotoxicity, which is a significant contributory cause of cancer, and protecting from carcinogens or toxic agents. They also were reported to display antiproliferative activity *in vitro* against breast [8]–[10], leukemia [8], [9], [11], hepatoma [9], [11]–[13], colon [11], [14]–[16], lung [9], prostate [9], ovarian [13], [17], and urinary bladder [10] cancer cells. However, the effect of rosemary on pancreatic carcinoma cells has not been reported to date. Regarding the tumor progression *in vivo*, the effect of rosemary extract in combination with an analogue of 1,25-dihydroxyvitamin D_3_ was assessed in a syngeneic mouse leukemia tumor model, and showed a strong cooperative antitumor effect [18].

Several rosemary components, such as carnosic acid [9], [11], carnosol [3], [11], [17], ursolic acid [3], as well as some of its essential oil constituents [13], have been proposed to be responsible for the anticancer effects of rosemary extracts. Although the concentration ratios of carnosol and carnosic acid were reported to influence the antioxidant and antimicrobial activities [19], the possible synergism of the rosemary components regarding the antitumor activity of rosemary extracts has not been reported yet.

Rosemary and its components were reported to modulate several pathways, such as those related to antioxidant response (e.g. glutathione metabolism [20] and Nfr2-dependent pathway [21]), AMPK and PPAR pathways [22], as well as apoptosis-related genes [17], but the molecular mechanism responsible for its antitumor effects is not completely understood yet. In order to properly apply rosemary as a nutritional supplement for cancer therapy, additional information regarding the most effective composition, its antitumor effect *in vivo* and its main molecular mediators is still needed. In this sense, we have previously reported the synergistic effect of the combination of supercritical fluid rosemary extract and 5-fluorouracil, the most commonly used drug in colon cancer therapy, through the modulation of TK1 and TYMS, which are enzymes related to the mechanism of action of this drug [16].

In this work, the antitumor activities of five carnosic-acid rich supercritical rosemary extracts (RE's) with different chemical composition have been assayed in colon and pancreatic cancer cells with the aim of determining the most potent RE and the different sensitivity among the cell lines, as well as the contribution of isolated components to the antitumor effect of the RE and the possible cooperative effect of their combination. Moreover, the effect of RE's on tumor progression *in vivo* has been assessed in colon cancer mouse xenografts. Furthermore, gene and miRNA expression analysis were studied after RE treatment in order to elucidate the molecular mechanism responsible for its antitumor activity both *in vitro* and *in vivo*.

## Materials and Methods

### 1. RE's and isolated compounds

RE's were obtained from *Rosmarinus officinalis* L. leaves as previously described in Vicente *et al*., 2013 [23]. Briefly, extractions were performed using a supercritical fluid pilot plant (Thar Technology, Pittsburgh, PA, USA, model SF2000). The temperature of the extraction cell and separators was maintained at 40°C, and the CO_2_ flow rate was 60 g/min. An amount of 0.5 kg of dried and grinded rosemary leaves were employed in each extraction assay. Different conditions (pressure, amount of ethanol as co-solvent and extraction time) were applied in order to obtain different RE's with increasing concentration of bioactive compounds (Table 1). Carnosic acid and carnosol were quantified by HPLC analysis, and essential oil components were determined by GC-MS analysis. The detailed methods for these determinations were previously described by Vicente *et al*. [23]. Carnosic acid (≥97.0% w/w) was purchased from TCI Europe (Belgium), and carnosol (≥98% w/w) was acquired from Sigma-Aldrich.

**Table 1:** Extraction conditions and chemical composition of supercritical fluid rosemary extracts (RE's).

Rosemary extract	Extraction and fractionation conditions	Carnosic acid (% w/w)	Carnosol (% w/w)	Main volatil compounds^a^ (% w/w)
RE-1	P = 300 bar, t = 360 min	10.89	1.05	12.79
RE-2	P = 300 bar. First step: fractionation^b^ during t = 60 min. Second step: t = 300 min without fractionation. Sample from separator 1.	16.90	1.90	13.59
RE-3	P = 150 bar, C = 10%(w/w), t = 180 min.	18.33	1.95	4.69
RE-4	P = 150 bar, C = 5%(w/w), t = 180 min.	25.66	3.81	10.42
RE-5	First step: P = 300 bar, t = 360 min. Second step: P = 150 bar, C = 10%(w/w), t = 180 min. Sample from second step.	30.69	2.58	2.04

a Borneol, bornyl acetate, camphor, 1,8-cineol and verbenone.

b Fractionation of the extracted material was performed by setting pressure of the first separator (S1) to 100 bar, while the second separator (S2) was maintained at the recirculation system pressure (50 bar).

P: extraction pressure, C: ethanol used as cosolvent (% w/w), t: extraction time.

### 2. Cell culture

Human colon (SW620 and DLD-1) cancer cells were obtained from American Type Culture Collection (ATCC, Manassas, VA, USA), and human pancreatic (MIA-PaCa-2 and PANC-1) cancer cells were acquired from Sigma-Aldrich. SW620, MIA-PaCa-2 and PANC-1 cells were cultured in DMEM, while DLD-1 cells were cultured in Roswell Park Memorial Institute (RPMI)-1640 medium. Both culture mediums were supplemented with 10% fetal bovine serum (FBS), 2 mmol/L glutamine, and 1% of antibiotic-antimycotic solution (containing 10 000 units/mL of penicillin base, 10 000 µg/mL of streptomycin base, and 25 000 ng/mL of amphotericin B)(Gibco, Grand Island, NY, USA). The cells were maintained under standard conditions of temperature (37°C), humidity (95%), and carbon dioxide (5%).

### 3. Cell viability assay

Cell viability was determined by the 3-(4,5-dimethyl-thyazol-2-yl)-2,5-diphenyl-tetrazolium (MTT) assay as previously described [16]. Briefly, 20 000–30 000 cells were seeded in 24-well plates, attached overnight, and treated with increasing concentrations of RE's. Following 48 h treatment, cells were incubated with MTT solution during 3 hours, and absorbance of the MTT metabolic product, which correlates with cell viability, was measured at 5601nm. At least three independent experiments were performed in quadruplicate. IC50 values (concentrations that caused 50% of cell viability inhibition) were calculated using a logistic regression.

### 4. Western blot analysis

Western blotting was performed to investigate the effect of RE's on the cleavage of PARP1 as previously described [16]. Briefly, cell proteins were separated by sodium-dodecyl-sulfate-PAGE electrophoresis, transferred to a nitrocellulose membrane and blocked with 5% BSA in TTBS buffer. Primary antibodies anti-PARP1 (BD Pharmingen) and anti-β-actin (Sigma-Aldrich) were used at 1/125 and 1/2 000, respectively, and anti-mouse IgG conjugated to horseradish peroxidase (GE Healthcare) was used at 1/40 000 as a secondary antibody to allow visualization by chemiluminescence using the Amersham ECL Prime WB Detection Reagent (GE Healthcare, Little Chalfont, UK). Molecular weights of protein bands were determined by the TotalLab software (TotalLab, Newcastle, UK). β-actin determination was used as an endogenous control of total protein quantity.

### 5. Tumorigenicity in nude mice

Female nude mice (Hsd∶Athymic Nude-*Foxn1nu*) of 6 weeks of age were purchased from Harlan Laboratories S.A. (Barcelona, Spain) for tumorigenicity assays. Xenografts of human colon cancer cells (SW620) were established by subcutaneously injecting 10^6^ cells suspended in 0.2 mL of 1∶1 mixture of DMEM and matrigel (BD Biosciences), as previously described [24]. Mice were then randomly divided into the groups (n = 8–10). RE was administered to test groups in the drinking water (1 mg RE/mL). The vehicle of the RE (absolute ethanol) was added to the drinking water of control groups at the same proportion as in the test groups (20 μL/mL). Tumor volume was measured twice a week during approximately 5 weeks. The experiment was approved by the Ethics Committee on Human and Animal Experimentation of the Biomedical Research Institute (CSIC-UAM, Madrid, Spain). Mice were kept in a pathogen-free housing (laboratory accreditation number: ES280790000188) with water and food *ad libitum*, 12-hour cycles of light/dark, and temperature maintained at 22±2°C in agreement with the Spanish institutional guidelines for the care and use of laboratory animals (RD 53/2013). In order to minimize animal suffering, behavior, mood and weight of animals were controlled to detect possible signs of toxicity, and a maximum tumor volume threshold of 1.2 cm^3^ was established to indicate the end of the experiment. Then, the animals were sacrificed with the use of CO_2_ (with gradual release of gas) in agreement with the European Directive 2010/63/UE and the Spanish guidelines (RD 53/2013). Afterwards, a sample of blood was collected in Sarstedt Multivette 600 EDTA tubes (Sarstedt), and centrifuged at 1500 g during 10 minutes for extraction of plasma samples, which were maintained at −80°C.

### 6. Total RNA extraction

RNeasy kit or miRNeasy kit (Qiagen, Valencia, CA, USA) were used according to the manufacturer's protocol to obtain total RNA or total RNA including miRNAs, respectively. The quantity and purity of the obtained total RNA samples were determined by UV-spectroscopy (NanoDrop 2000 Spectrophotometer, Thermo Fisher Scientific, Waltham, MA, USA). For total RNA (including miRNAs) extraction from mouse plasma samples, the protocol was modified as follows: 500 μL of RWT buffer was used at first washing, RPE buffer washing was performed three times, and column containing the RNA was then centrifuged at 15 000 g during 2 minutes and air-dried during 1 minute before RNA elution.

### 7. Gene expression analysis

Microarray hybridization was commissioned from NIMgenetics S.L. (Madrid, Spain). RNA quality control was performed in a 2100 Bioanalyzer (Agilent Technologies, Santa Clara, CA, USA), obtaining RNA integrity numbers (RIN) ≥8. Complementary RNAs were prepared for hybridization in an Agilent G4112F platform (Whole Human Genome Microarray 44k) using the One-Color gene expression system (Agilent Technologies) according to the manufacturer's instructions. Microarray data were extracted with Feature Extraction Software version 10.7 (Agilent Technologies), and expression data analysis was carried out with Babelomics (http://babelomics.bioinfo.cipf.es) [25], using the Agilent one-channel workflow. The background correction method selected was the one specific for Agilent arrays, and normalization was performed by using the quantile normalization method. Differential expression was assessed by using the LIMMA method. Benjamini and Hochberg false discovery rate (FDR) was applied for multiple test correction, and an adjusted p-value <0.05 was considered as statistically significant. An additional cutoff threshold of 50% change in gene expression was used to define a gene as being differentially regulated. Complete results of microarray analysis can be found in Gene Expression Omnibus (*GEO*) database (accession number: GSE56496).

Individual gene expression analysis was performed by quantitative RT-PCR (qRT-PCR). To that end, 400 ng RNA was reverse-transcribed to cDNA using the High Capacity RNA-to-cDNA Master Mix system (Applied Biosystems, Carlsba, CA, USA), as directed by the manufacturer. For gene expression analysis of GCNT3, we used the specific Taqman gene expression assay Hs01921181_s1 (Applied Biosystems). qRT-PCR was performed in the 7900HT Real-Time PCR System (Applied Biosystems), according to the manufacturer's protocol. 18S expression in each sample was used as endogenous control (specific assay Hs99999901_s1, Applied Biosystems). qRT-PCR data extraction was performed using the RQ Manager software (Applied Biosystems), and the 2^−ΔΔCt^ method was used to calculate the relative expression of each gene (RQ = 2^−ΔΔCt^) as previously described [26].

### 8. miRNA expression analysis

The screening of the modulation of 754 different miRNAs by RE was carried out by using the TaqMan Array Human MicroRNA A+B Cards Set v3.0 (Applied Biosystems). To that end, miRNA reverse transcription was performed with the TaqMan miRNA Reverse Transcription kit and the Megaplex Primer Pools, Human Pools set v3.0. Afterwards, qRT-PCR was performed with the cards set and the TaqMan Universal PCR Master Mix No AmpErase UNG (Applied Biosystems) in the 7900HT Real-Time PCR System (Applied Biosystems), as directed by the manufacturer, and analyzed by the 2^−ΔΔCt^ method [26]. In order to determine the miRNAs that target GCNT3 gene according to *in silico* analysis, we checked four different databases (miRanda, TargetScan, Diana-microT, and PITA) and mirSVR score values from http://www.microrna.org/microrna/getDownloads.do.

To determine the expression of individual miRNAs, the specific TaqMan miRNA assay (ref. 000390 for miR-15b, and ref. 002182 for miR-939) was used according to the manufacturer's protocol. Retrotranscription of each miRNA was performed with TaqMan miRNA Reverse Transcription Kit (Applied Biosystems) and the specific primers and TaqMan Universal PCR Master Mix II no UNG was used for the qRT-PCR, which was performed in the 7900HT Real-Time PCR System (Applied Biosystems).

### 9. Statistical analysis

Data are presented as mean ± SEM. Comparisons between two groups were analyzed by Student's t test or the non-parametric alternative Mann-Whitney U test according to normality of data distribution. The statistically significant differences among several groups were determined by one-way ANOVA with Bonferroni's *post hoc* test. The statistical test used in each experiment is indicated in the corresponding figure legend. Tumor volume comparisons between control and treated groups were analyzed using repeated measures ANOVA and Bonferroni's test. Statistically significant values are indicated by asterisks as follows: *p≤0.05; **p≤0.01; ***p≤0.001. IBM SPSS Statistics version 20 (SPSS Inc., Chicago, IL) was used for statistical analysis.

## Results

### 1. RE's obtained by supercritical fluid technology

*Rosmarinus officinalis* L. leaves were used as starting material for supercritical fluid extraction by applying different conditions of pressure, percentage of ethanol as co-solvent, and extraction time (Table 1). Five RE's with different composition and antioxidant activity were obtained, and used to examine their antitumor activities.

### 2. Differential cell viability inhibitory activity of supercritical RE's on colon and pancreatic cancer cells

The effect of the five different RE's obtained by supercritical fluid technology on cell viability of colon (SW620 and DLD-1) and pancreatic (PANC-1 and MIA-PaCa-2) cancer cells was assessed by the MTT assay. The IC50 values of each RE in the different cell types were calculated in order to compare the potency of the RE's in each cell line, and the sensitivity of the cell lines to each RE. The results indicated that PANC-1 (pancreatic cancer) is the most resistant cancer cell line to the effect of the RE's, followed by MIA-PaCa-2 (pancreatic cancer), and colon cancer SW620 and DLD-1 cell lines, which are the most sensitive. Therefore, RE's appear to be more effective in inhibiting cell viability of colon cancer cells in comparison to pancreatic cancer cells.

**Figure 1:**
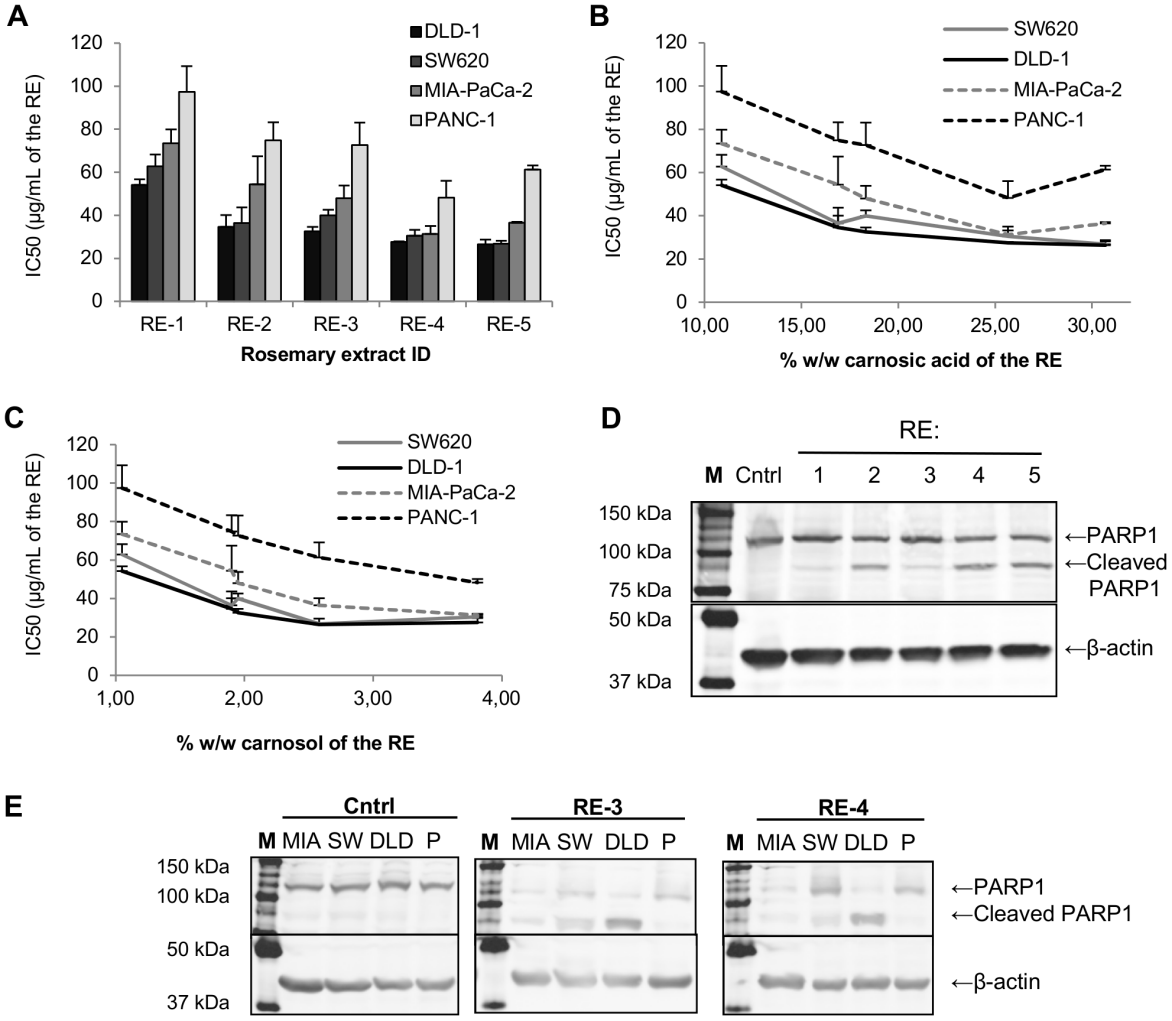
Viability inhibition and death induction of colon and pancreatic cancer cells by five different RE's. Colon (SW620 and DLD-1) and pancreatic (MIA-PaCa-2 and PANC-1) cancer cells were treated with increasing concentrations of five different RE's during 48 h and cell viability was determined by MTT assay. (**A**) Bars represent the IC50 values (50% cell viability inhibition) of the five different RE's in each cancer cell line. (**B-C**) IC50s of the five RE's in the four cancer cell lines plotted against the carnosic acid (**B**) or carnosol (**C**) content of the RE's. IC50 values are expressed as the mean ± SEM of at least three independent experiments, each performed in quadruplicate. (**D-E**) PARP1 cleavage induced by different RE's in colon and pancreatic cancer cells after 48 h treatment. (**D**) Colon cancer cells (DLD-1) were treated with 70 µg/mL of five different RE's (RE-1 to RE-5) or vehicle (Cntrl). (**E**) Two colon (DLD-1 and SW620) and two pancreatic (MIA-PaCa-2 and PANC-1) cancer cell lines were treated with 110 µg/mL of RE-3, 90 µg/mL of RE-4 or vehicle (Cntrl). Western blot figures show representative results of three independent experiments. DLD: DLD-1, M: marker of molecular weight, MIA: MIA-PaCa-2, P: PANC-1, RE: supercritical rosemary extract, SW: SW620.

Regarding the different RE's, RE-1 is the less potent, while RE-4 and RE-5 are the most potent inhibitors of colon and pancreatic cancer cell viability (Figure 1A). RE-2 and RE-3 show similar effects, which are intermediate between that of RE-1 and RE's 4 and 5. Although RE-5 possesses a higher content in carnosic acid than RE-4, its effect on cell viability is similar or even lower than that of RE-4. This difference might be attributed to the higher content of carnosol and/or essential oil of RE-4 in comparison with RE-5. Interestingly, while RE-4 activity in colon cancer cells is similar to that of RE-5, pancreatic cancer cells appear to be more sensitive to RE-4 than to RE-5. This could be explained by a different sensitivity of pancreatic cancer cells to carnosol and/or essential oil in comparison to colon cancer cells since the ratios carnosol∶carnosic acid and essential oil∶carnosic acid are higher in RE-4 than in RE-5 (see Table 1). Moreover, a synergistic effect of the combination of carnosic acid and carnosol or volatile compounds might explain the differential effect of these two RE's. The potency of the RE's (IC50) was plotted against carnosic acid or carnosol content (both previously reported to be responsible for the antitumor activities of rosemary) in order to observe the correlations between these characteristics and the effect of the RE's on cell viability. The plot against carnosic acid (Figure 1B) shows a correlation between this compound and cell viability inhibition until RE-4 (25.66% w/w carnosic acid), but RE-5, with a higher carnosic acid content (30.69% w/w), shows a similar effect on colon cancer cells and even a lower effect on pancreatic cancer cells in comparison with RE-4. On the contrary, the plot against carnosol content (Figure 1C) shows a good correlation in pancreatic cancer cells, while the effects of RE's 4 and 5, although the former contains approximately 50% more carnosol than the latter, is very similar in colon cancer cells. Therefore, despite both carnosol and carnosic acid show a relatively good correlation with the cell viability inhibitory effect of the RE's, none of these compounds on its own completely explain their different antitumor potency.

### 3. Differential induction of apoptosis by five different RE's in colon and pancreatic cancer cells

In order to compare the induction of cell death on colon and pancreatic tumor cells by the different RE's we analyzed PARP1 cleavage by western blot. On the one hand, the same cell line (DLD-1) was treated with the different RE's at the same concentration, in order to assess their different potency in inducing tumor cell death. Figure 1D indicates that the five RE's are able to induce tumor cell death at 70 µg/mL, although RE-1 is the less active and RE's 4 and 5 are the most active extracts in relation to this effect. This result is also consistent with the results obtained in cell viability experiments, confirming the lowest activity of RE-1 and the highest activity of RE-4 and RE-5 in both inhibiting cell viability and inducing death of cancer cells.

On the other hand, colon (DLD-1 and SW620) and pancreatic (PANC-1 and MIA-PaCa-2) cancer cells were treated with the same concentration of RE-3 or RE-4 to determine the sensitivity of the different cell lines to the RE's. As it can be observed in Figure 1E, colon cancer DLD-1 cell line is the most sensitive while pancreatic cancer PANC-1 cell line is the less sensitive to the cell death induction by both RE's. This is consistent with the results previously obtained in cell viability, and confirms the different sensitivity of the colon and pancreatic cancer cell lines to the antitumor effects of the RE's.

### 4. Carnosic acid antitumor effect on colon and pancreatic cancer cells is significantly enhanced by minimal concentrations of carnosol

**Figure 2:**
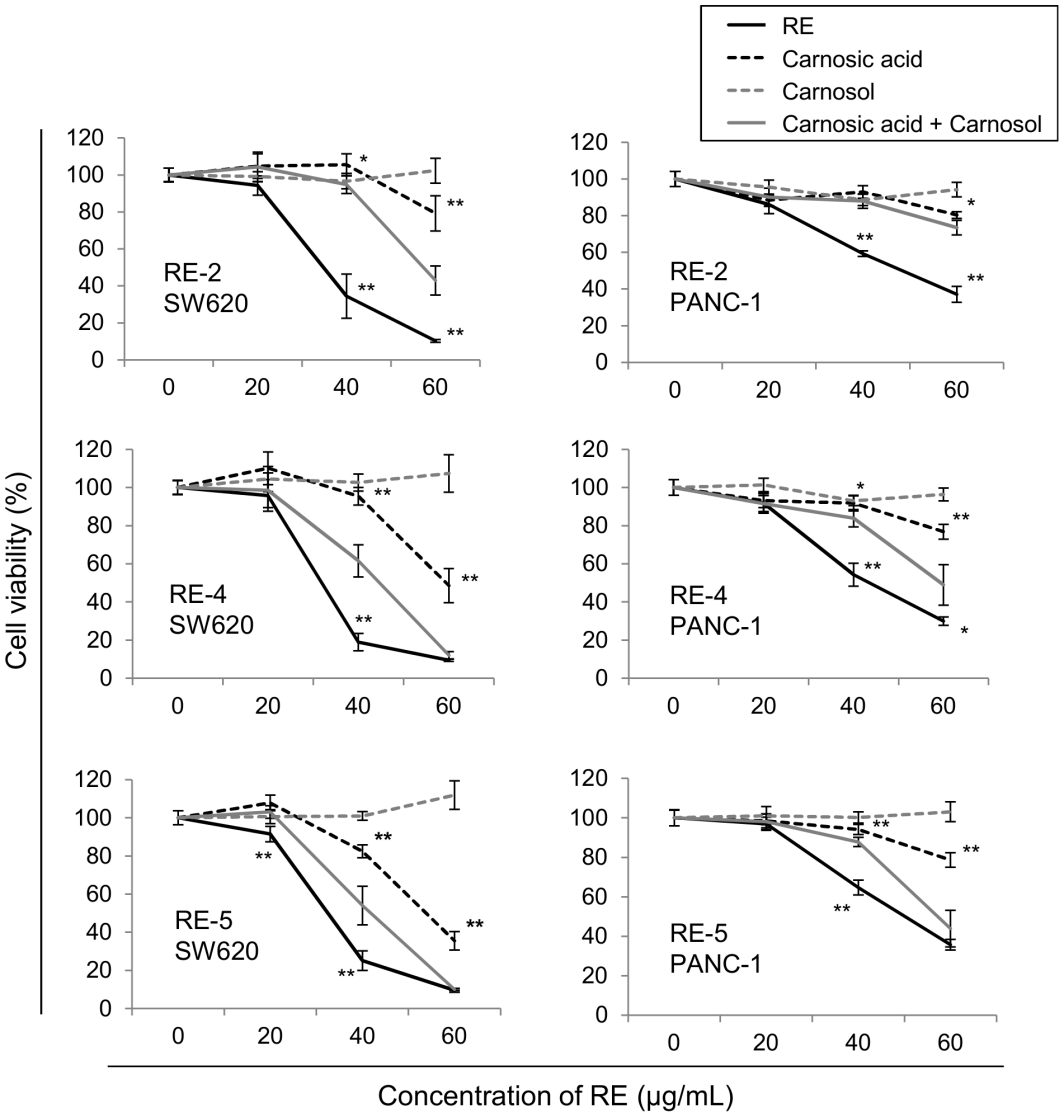
Inhibition of cancer cell viability by different RE's in comparison with their major active components. Cell viability inhibition of cancer cells by different RE's and their equivalent concentrations of carnosic acid alone, carnosol alone, or both compounds in combination. SW620 colon and PANC-1 pancreatic cancer cells were treated with increasing concentrations of RE-2, RE-4 and RE-5, and the same concentrations of carnosic acid, carnosol, and the combination of both present in the RE's at each assayed condition. Extract ID and tumor cell line used in the assay are indicated in each graph. Results are shown as the mean ± SEM of three independent experiments, each performed in quadruplicate. Asterisks indicate statistically significant differences of carnosic acid or extract in comparison with the combination of carnosic acid and carnosol (U de Mann-Whitney; *p≤0.05; **p≤0.01). RE: supercritical rosemary extract.

With the aim of determining which is the contribution of the content of carnosic acid and carnosol to the antitumor activity of the RE's, and thus explain the different activities of the five RE's, we assayed the effect on cell viability of the different RE's in comparison with the effect of the same concentrations of carnosic acid and carnosol present in the RE, both separately and in combination, in colon (SW620) and pancreatic (PANC-1) cancer cells (Figure 2). We observed that the concentrations of carnosic acid found in RE's inhibited tumor cell viability in a dose-dependent manner, while carnosol at those concentrations did not show any effect on cell viability. Surprisingly, carnosol at these inactive concentrations significantly potentiated the activity of carnosic acid. Therefore, the combination of carnosic acid and carnosol found in the three RE's is more effective than the carnosic acid alone, even though the low concentration of carnosol present in the RE is ineffective on its own. However, the effect of the RE is significantly higher than the combination of the equivalent concentrations of carnosol and carnosic acid, indicating that additional components of the RE's (e.g. volatile oil compounds) contribute to their inhibitory effect on tumor cell viability.

### 5. Oral intake of RE's inhibits tumor growth of colon cancer xenografts in nude mice

**Figure 3:**
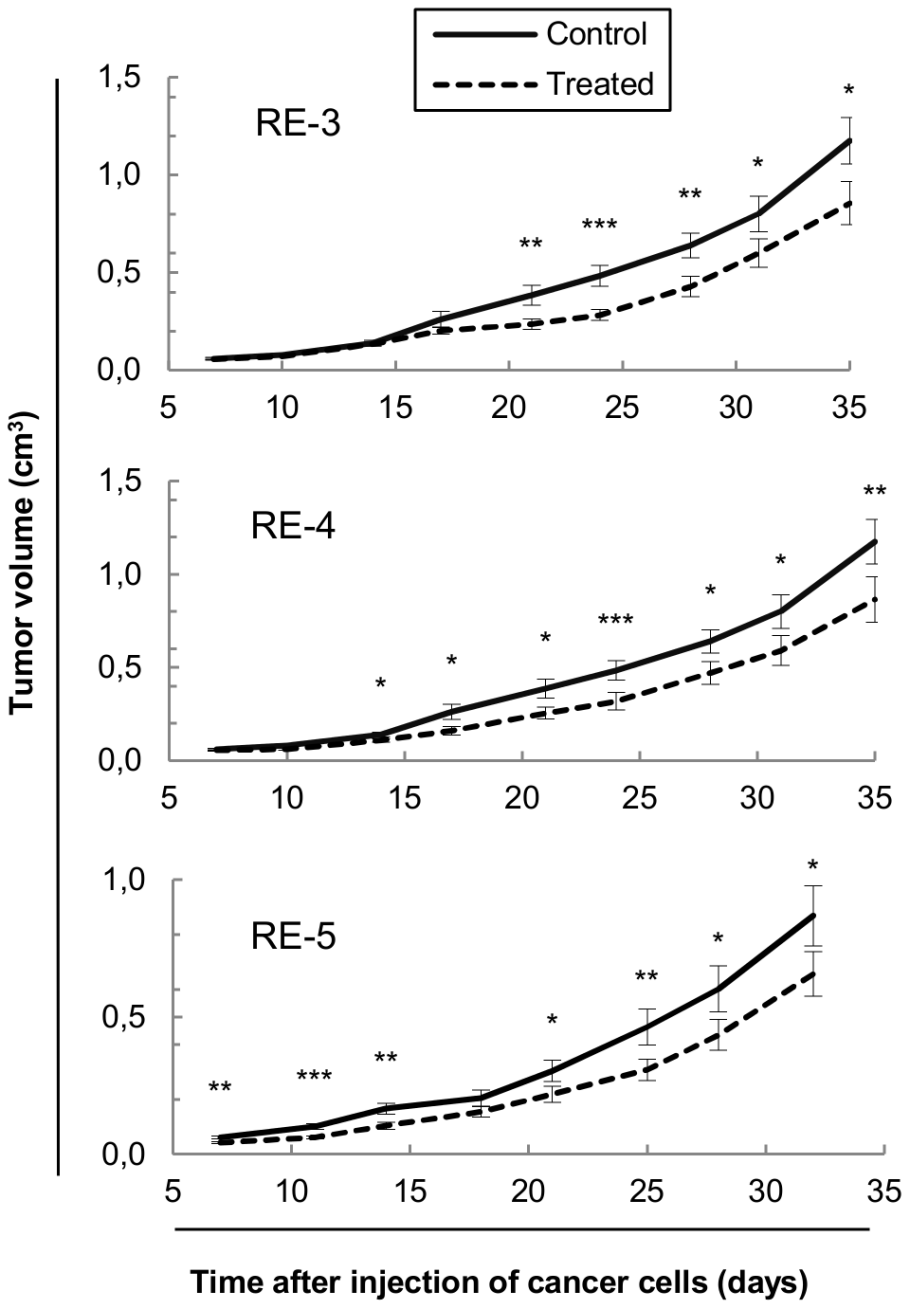
Antitumorigenic activity of different RE's in human colon cancer xenografts. RE (test groups) or vehicle (control groups) was administered to nude mice bearing SW620 colon cancer xenografts in the drinking water (1 mg RE and 20 μL ethanol as vehicle per mL of drinking water). Three RE's with different composition (RE-3, RE-4 and RE-5, indicated in each graph) were assayed. Tumor volumes were monitored twice a week during 32–35 days. Results are shown as mean ± SEM (n = 16–20) and repeated measures ANOVA with Bonferroni's test was used to determine the statistically significant differences between treated and control groups (*p≤0.05; **p≤0.01; ***p≤0.001). RE: supercritical rosemary extract.

In order to assay the effect of the RE's on tumor progression *in vivo* we administered RE-3, RE-4 or RE-5 in the drinking water to nude mice bearing human colon cancer xenografts. The results indicated that the three different RE's assayed possess the ability to significantly inhibit tumor volume *in vivo* (Figure 3). Since the tumor cells were already present in the organism when the treatment with RE started, these results suggest that RE's might not only act as a putative chemopreventive agent as previous works indicate, but also as an effective agent in blocking tumor development and progression. We did not detect any significant change in the behavior, mood and weight of the animals along the duration of the experiment, indicating the lack of toxicity of the RE's under these conditions of administration. Although the volume inhibition at the end of the experiment caused by the different RE's was very similar (27.2%, 26.4% and 24.4% by RE-3, RE-4 and RE-5, respectively), the effect appeared later in RE-3 in comparison with RE-4 and RE-5, which is consistent with the lower activity of RE-3 observed *in vitro* in comparison with RE-4 and RE-5.

### 6. Antitumor effect of RE's correlates with up-regulation of glycosyltransferase GCNT3 gene expression

**Figure 4:**
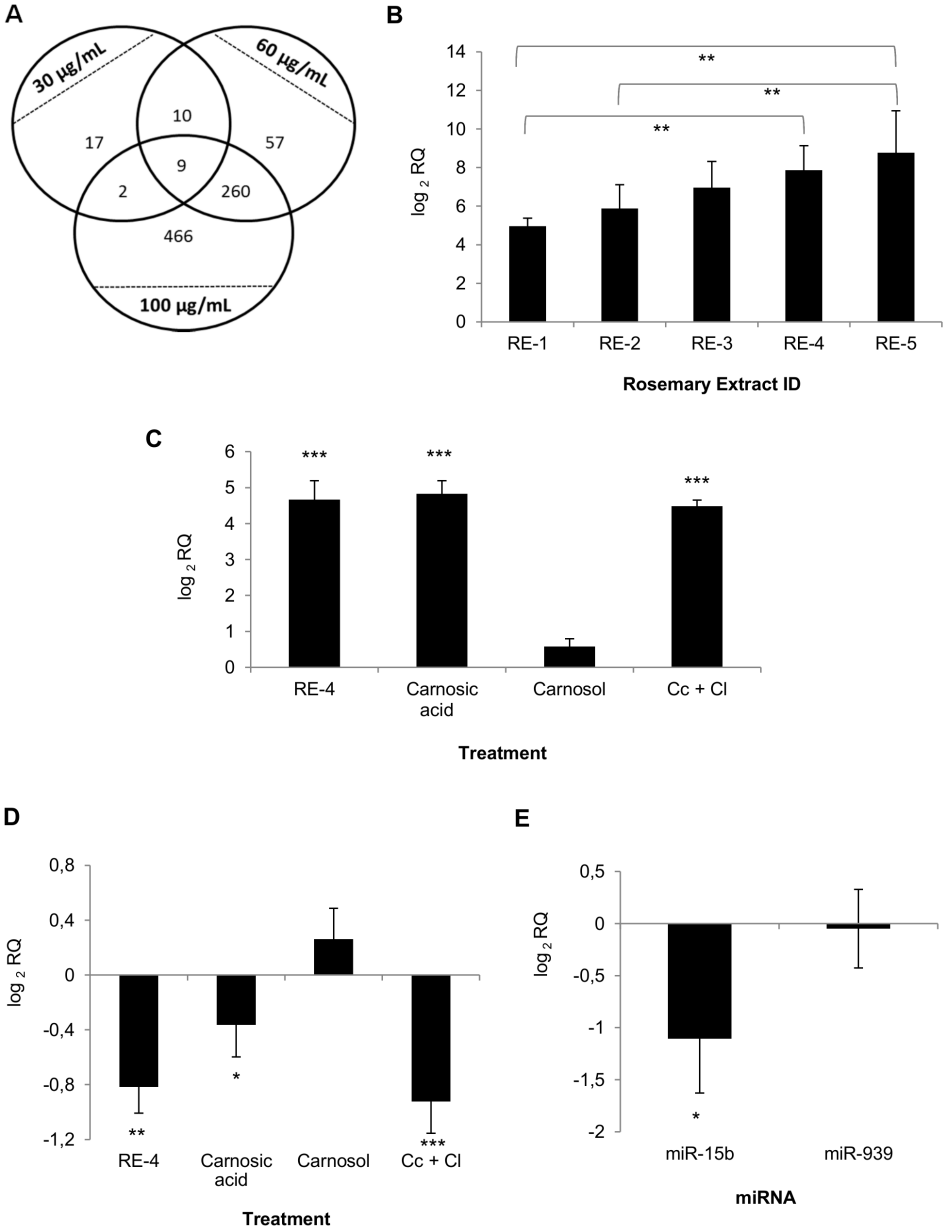
Modulation of gene and miRNA expression by RE. (**A**) Venn diagram representing overlap of the genes significantly modulated (p<0.05 and fold change (FC) >±50%) by RE-2 at 30, 60 and 100 µg/mL after 48 h treatment in SW620 cells according to microarray data. (**B**) Up-regulation of GCNT3 gene expression in SW620 colon cancer cells by five RE's with different composition (RE-1, RE-2, RE-3, RE-4 and RE-5) at the same concentration (90 µg/mL) during 48 h. Bars represent the mean ± SEM of two independent experiments, each performed with biological triplicates and technical duplicates. ANOVA with Bonferroni's *post hoc* test was applied to test statistically significant differences among RE's. (**C**) Up-regulation of GCNT3 gene expression in SW620 colon cancer cells by 48 h treatment with RE-4 at 60 µg/mL and carnosic acid, carnosol, and the combination of both compounds at the concentrations present in this RE. Bars represent the mean ± SEM of two independent experiments, each performed with biological triplicates and technical duplicates. Student's t test was applied to determine the statistically significant differences between treated and control cells. (**D**) Down-regulation of miR-15b gene expression in SW620 colon cancer cells by 48 h treatment with RE-4 at 60 µg/mL and carnosic acid, carnosol, and the combination of both compounds at the concentrations present in this RE. Student's t test was used to test statistically significant differences between treated and control cells. (**E**) Modulation of miR-15b and miR-939 (as a negative control) in plasma of colon cancer xenograft nude mice after orally intake of RE-5 (1 mg/mL in drinking water), in comparison with the control group of mice. Bars represent mean ± SEM (n = 8). Student's t test was used to assess statistically significant differences between treated and control groups. * p≤0.05, ** p≤0.01, *** p≤0.001. RE: supercritical rosemary extract, Cc+Cl: Carnosic acid and carnosol.

In order to gain an insight into the antitumor mechanism of action of RE's, microarray analysis was performed. To that end, RNA was extracted from SW620 cells treated with increasing concentrations of RE-2. The numbers of genes whose expression was significantly altered in comparison with the control (p<0.05 and ±50% expression change) are shown in Figure 4A, and genes modulated at the three concentrations assayed are presented in Table 2. On the basis of its dose-dependent induction and its reported activity as tumor suppressor [27], GCNT3 was selected as one of the genes potentially involved in the antitumor mechanism of action of RE. On the other hand, the analysis of the expression of miRNAs targeting GCNT3 showed that miR-15 family is significantly down-regulated by RE-2 (Table 3). Moreover, GCNT3 expression was analyzed in colon cancer cells treated with the five different RE's at the same concentration (90 µg/mL). The results indicate that over-expression levels of GCNT3 increases from RE-1 to RE-5 (Figure 4B), thus correlating with the potency of these RE's to inhibit cell viability and induce cell death of colon cancer cells. As it can be observed, carnosic acid is the main responsible for the induction of GCNT3 expression (Figure 4C) and miR-15b down-regulation (Figure 4D) by RE, while carnosol did not show a significant effect in the modulation of either GCNT3 or miR-15b at the concentrations found in the RE.

**Table 2:** Modulation of gene and miRNA expression by RE-2 in colon cancer cells.

	30 µg/mL		60 µg/mL		100 µg/mL	
HGNC.Symbol	p value	FC^a^	p value	FC^a^	p value	FC^a^
FUT3	0.00807	1.59	0.00172	1.59	0.00009	2.03
PLCB2	0.02310	2.16	0.01838	1.86	0.00092	2.56
CLIP4	0.00102	1.98	0.00008	2.17	0.00002	2.25
GCNT3	0.00654	1.54	0.00009	2.38	0.00002	2.49
CBR3	0.03568	1.61	0.00056	2.50	0.00008	2.88
AKR1B10	0.00045	2.27	0.00003	2.68	0.00001	2.46
BEST1	0.04765	2.05	0.00191	2.97	0.00175	2.58
CSTA	0.00455	2.49	0.00020	3.40	0.00006	3.32
DEFB103B	0.02419	2.07	0.00014	4.78	0.00003	5.18

Microarray data of significantly modulated genes in SW620 colon cancer cells after treatment during 48 h with three different concentrations (30, 60 and 100 µg/mL) of RE-2.

a FC: Fold change.

**Table 3:** Modulation of miRNA expression by RE-2 in colon cancer cells.

miRNA	SUM^a^	log RQ mean	log RQ SD	mirSVR score^b^	PhastCons score^c^
hsa-miR-15a	4	−3.50	0.29	−0.4521	0.467
hsa-miR-15b	4	−0.54	0.18	−0.5344	0.5108
hsa-miR-28-5p	4	−0.52	0.25	−1.0479	0.5907
hsa-miR-330-3p	3	−0.34	0.04	−0.0218	0.5641
hsa-miR-27b	2	−0.50	0.23	−0.0196	0.5451
hsa-miR-454*	2	−0.75	0.05	−0.9911	0.5451

Modulation of the expression of miRNAs targeting GCNT3 in SW620 colon cancer cells by RE-2 at 60 µg/mL during 48 h according to qRT-PCR data.

a SUM: number of databases that report the interaction of GCNT3 and the miRNA according to *in silico* analysis.

b mirSVR score: likelihood of mRNA downregulation by the miRNA. It is calculated by a regression model based on the sequence and contextual features of the predicted miRNA-mRNA duplex. The lower the mirSVR score, the higher the likelihood of mRNA downregulation.

c PhastCons score: indicates the probability of negative selection and range between 0 (no conservation) and 1 (perfect conservation).

### 7. Non-invasive monitoring of RE antitumor effect by determining circulating miR-15b

Analysis of circulating miRNAs in plasma of mice with colon cancer xenografts after orally intake of RE-5 (1 mg/mL in drinking water) revealed that miR-15b is significantly down-regulated by rosemary in comparison with the control group of mice (Figure 4E), in agreement with the results observed *in vitro*. The specificity of the down-regulation of miR-15b in mouse plasma was assessed by the parallel analysis of miR-939 expression, which was used as a negative control. This result suggests that circulating miR-15b levels, which analysis requires minimal invasive methods, might be used to monitor the antitumor effect of RE *in vivo*.

## Discussion

Scientific literature reflects antitumor effects of several rosemary extracts mainly on skin [3], breast [4], colon [16] and leukemia [18], among other cancer types. Moreover, some rosemary components such as carnosic acid and carnosol have been reported to provide the antioxidant [19] and anticancer [11] effects to rosemary extracts. However, the possible synergistic effect of the combination of rosemary components has not been addressed yet. In this work, we assayed the antitumor effect of several supercritical RE's with different composition on colon and pancreatic cancer cells, in terms of cell viability and cell death. To our knowledge, neither RE's nor their main components have been assayed to determine their antitumor activities in pancreatic carcinoma to date. The results on cell viability show that the sensitivity of tumor cells to the different RE's is (from less sensitive to more sensitive): PANC-1 (pancreas), MIA-PaCa-2 (pancreas), SW620 (colon) and DLD-1 (colon). The results on apoptosis induction also suggest that colon cancer cells might be more sensitive than pancreatic cancer cells to the antitumor effects of the extracts. Thus, the different potency of the RE's previously observed in cell viability by MTT assay correlates with their potency in inducing cell death assessed by western blot analysis of PARP1 cleavage. Future work is needed in order to find the cellular or molecular characteristics that make PANC-1 cells especially resistant to the antitumor effects of rosemary extracts.

Several advantages warrant the use of rosemary extracts, and specifically supercritical extracts, instead of their isolated components in cancer treatment. On the one hand, the obtaining of rosemary extracts is much less expensive than the isolation of its compounds. Moreover, supercritical rosemary extracts do not contain chemical residues, which would entail harmful effects. Indeed, supercritical fluid rosemary extract has been recognized as a healthy component by the European Food Safety Authority (EFSA), and is currently used as an antioxidant food additive [28]. On the other hand, as we have demonstrated in this work the combination of carnosol and carnosic acid exerts a higher effect than the sum of their effects separately. Jordan *et al.* recently reported the relationship between the carnosic acid and carnosol content of RE's with their antimicrobial and antioxidant activities [19]. They found that the two diterpenes equally affected to the antioxidant activity, whereas antimicrobial effect was higher when the carnosol∶carnosic acid ratio increased. Future studies are warranted in order to determine the specific carnosol∶carnosic acid ratio needed to achieve the highest antitumor effect. Moreover, additional rosemary components (e.g. volatile compounds) further increase the antiproliferative effect of RE, thus contributing to the higher antitumor activity of the extract in comparison to the effect of the combination of its main components (carnosic acid and carnosol).

In order to test the efficacy of RE *in vivo*, several RE's were orally administered to mouse colon cancer xenografts. Previous animal studies found in the literature showed the preventive effect or rosemary extract *in vivo* in several tumor types. In our experiment, since the tumor cells are already in the organism at the time of treatment, we demonstrate the inhibitory effect of rosemary extract on tumor progression *in vivo*. The activities of the three RE's assayed (RE-3, RE-4 and RE-5) resulted very similar at the end of the experiment despite cell viability *in vitro* experiments showed the lesser efficacy of RE-3. However, the effect of RE-3 began later than those of RE-4 and RE-5. One explanation could be the saturation of the absorption mechanisms of rosemary active components in the bowel, but more experiments are needed to address this issue.

Rosemary and their components were reported to modulate glutathione metabolism [20], Nfr2-dependent pathway [21], AMPK and PPAR pathways [22], among others, as well as apoptosis-related genes [17]. However, the antitumor mechanism of action is not completely understood. In this study, we observed the up-regulation of GCNT3 by RE, and the correlation of this up-regulation with its antitumor efficacy. The rosemary component responsible for this modulation is carnosic acid. Since GCNT3 has been previously reported to possess tumor suppressor activities in colon cancer [27], it is up-regulated by several chemotherapeutic drugs and its overexpression correlates with a better outcome of colon cancer patients (González-Vallinas M *et al*., submitted for publication) we propose that GCNT3 may be a key molecule in the antitumor action of rosemary.

The finding that miR-15b, which was reported to target GCNT3 by *in silico* analysis, correlated with the antitumor effect of rosemary and can be found in mouse plasma, provides a potential suitable biomarker to monitor the *in vivo* response to RE. In addition, miR-15b has been recently proposed as potential biomarker for colorectal cancer since it has been found up-regulated in colorectal cancer patients, which suggests a relevant role of this miRNA in the progression of the disease [29]. The determination of the circulating miRNAs is a non-invasive and relatively accessible method which could be useful in order to discriminate the responder and non-responder individuals during the treatment. Future work should be directed both to analyze the functional role of these molecules in the action of this agent, and to investigate their clinical value as potential molecular biomarkers.

## Conclusions

In summary, our results indicate that RE exerts antitumor activity on both colon and pancreatic cancers, probably through the up-regulation of GCNT3 and the down-regulation of miR-15b, and constitutes a promising therapeutic tool in the treatment of patients suffering from these diseases. Moreover, down-regulation of plasmatic miR-15b levels is proposed as a potential non-invasive biomarker to monitor the anticancer effect produced by RE.
